# MicroRNAs as the critical regulators of cisplatin resistance in gastric tumor cells

**DOI:** 10.1186/s41021-021-00192-4

**Published:** 2021-06-07

**Authors:** Amir Sadra Zangouei, Meysam Moghbeli

**Affiliations:** 1 Student Research Committee, Faculty of Medicine, Mashhad University of Medical Sciences, Mashhad, Iran; 2 Department of Medical Genetics and Molecular Medicine, School of Medicine, Mashhad University of Medical Sciences, Mashhad, Iran

**Keywords:** MicroRNA, Cisplatin, Multi drug resistance, Chemo resistance, Cancer

## Abstract

Combined chemotherapeutic treatment is the method of choice for advanced and metastatic gastric tumors. However, resistance to chemotherapeutic agents is one of the main challenges for the efficient gastric cancer (GC) treatment. Cisplatin (CDDP) is used as an important regimen of chemotherapy for GC which induces cytotoxicity by interfering with DNA replication in cancer cells and inducing their apoptosis. Majority of patients experience cisplatin-resistance which is correlated with tumor metastasis and relapse. Moreover, prolonged and high-dose cisplatin administrations cause serious side effects such as nephrotoxicity, ototoxicity, and anemia. Since, there is a high rate of recurrence after CDDP treatment in GC patients; it is required to clarify the molecular mechanisms associated with CDDP resistance to introduce novel therapeutic methods. There are various cell and molecular processes associated with multidrug resistance (MDR) including drug efflux, detoxification, DNA repair ability, apoptosis alteration, signaling pathways, and epithelial-mesenchymal transition (EMT). MicroRNAs are a class of endogenous non-coding RNAs involved in chemo resistance of GC cells through regulation of all of the MDR mechanisms. In present review we have summarized all of the miRNAs associated with cisplatin resistance based on their target genes and molecular mechanisms in gastric tumor cells. This review paves the way of introducing a miRNA-based panel of prognostic markers to improve the efficacy of chemotherapy and clinical outcomes in GC patients. It was observed that miRNAs are mainly involved in cisplatin response of gastric tumor cells via regulation of signaling pathways, autophagy, and apoptosis.

## Background

Gastric cancer (GC) is the 6th frequent and 2nd cancer related mortality globally [1]. Surgery is considered as the primary therapy in early stage GC, while majority of the patients are diagnosed in advanced stages of tumor progression that needs chemotherapeutic treatment. The combination of surgery along with chemotherapy is considered a curative approach for treating patients with gastric cancers, leading to higher disease-free survival and reduced risk of tumor relapse and metastasis [2, 3]. Despite the declining trend of GC incidence during recent decades, it still remains as one of the leading cause of cancer related mortalities globally [1]. Apart from fundamental diagnostic and therapeutic advances during recent decade, there is still a high ratio of poor prognosis among GC patients [4]. Surgery and chemotherapy can improve GC survival by 10–15 % [2, 3]. Combined chemotherapeutic treatment is the method of choice for advanced and metastatic gastric tumors. Cisplatin (CDDP) is used as an important regimen of chemotherapy for GC which functions through induction of DNA adducts formation in nucleus and mitochondria [5, 6]. Cisplatin induces cytotoxicity by interfering with DNA replication in cancer cells and inducing their apoptosis. Nevertheless, a majority of patients develop cisplatin-resistance which has been shown to be correlated with cancer metastasis and relapse [7]. Moreover, prolonged and high-dose cisplatin administrations cause serious side effects such as nephrotoxicity, ototoxicity, and anemia [8]. Therefore, it is important to enhance the sensitivity of GC cells to cisplatin in order to maximize the efficacy of chemotherapy for the chemo resistant patients. Since, there is a high rate of recurrence after CDDP treatment in GC patients; it is required to clarify the molecular mechanisms associated with CDDP resistance to introduce novel therapeutic methods. Various mechanisms are involved in drug resistance including drug efflux, reduced apoptosis, increased DNA repair ability, and drug detoxification [9]. Cisplatin accumulates in the mitochondria and causes mitochondrial dysfunctions resulting in the induction of cell apoptosis and oxidative/endoplasmic reticulum stress [10]. Due to the absence of appropriate early detection methods, most GC cases are diagnosed at late stages when the treatment is not effective [11]. About 70 % of GC patients are diagnosed with middle-advanced stage and show a 5-year survival rate of less than 20 % [12].

**Fig. 1:**
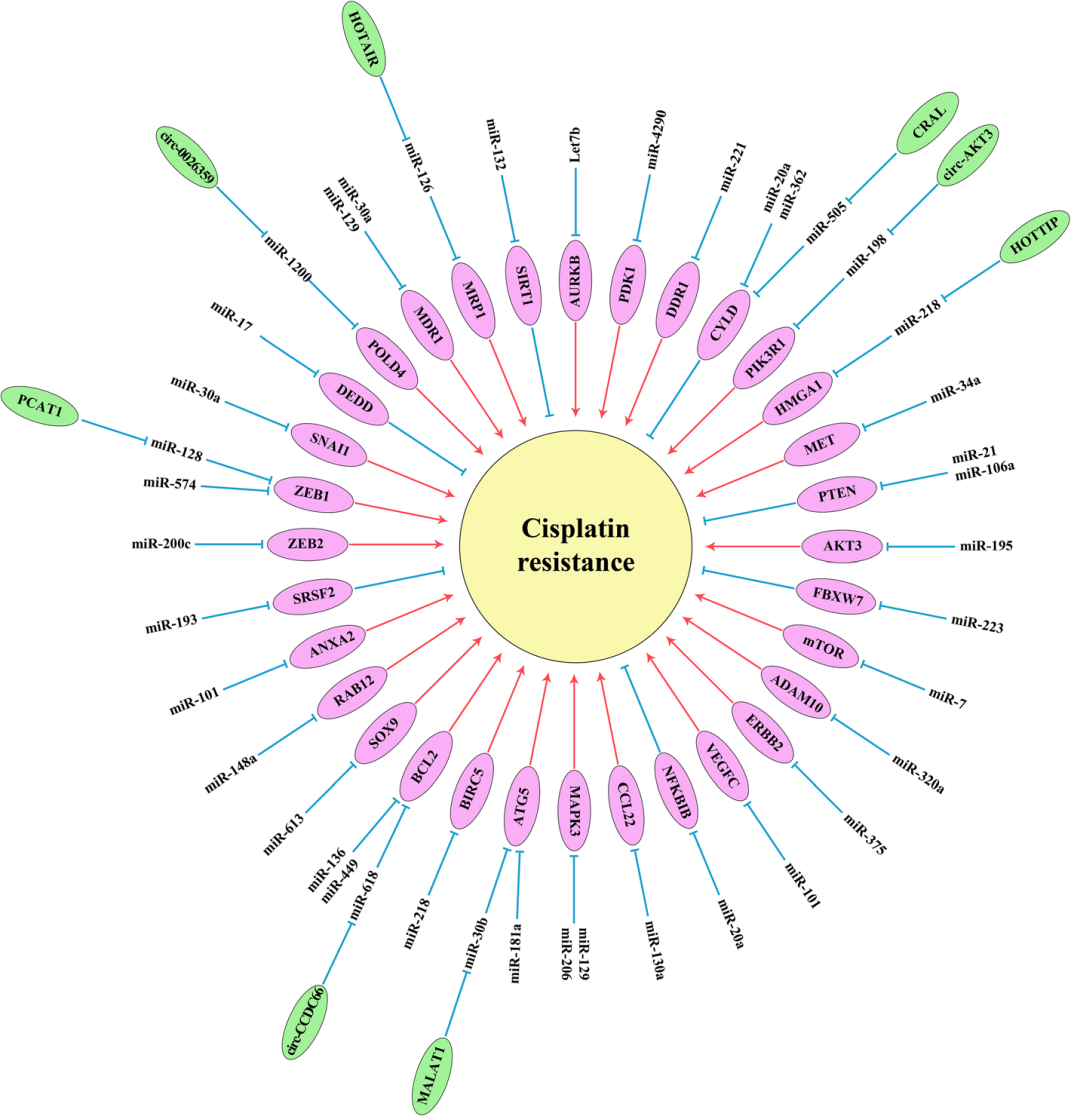
Molecular mechanisms of miRNAs associated with cisplatin resistance in gastric tumor cells

MicroRNAs (miRNAs) are small non-coding RNAs (∼22 nucleotides) involved in regulation of their target mRNAs through translation suppression or degradation [13]. MiRNAs have key roles in regulation of cellular mechanisms such as proliferation, differentiation, and apoptosis. Aberrant expression of miRNAs is associated with the development of chemo resistance [14]. Therefore, in present review we have summarized all of the miRNAs associated with cisplatin resistance to pave the way of introducing a miRNA-based panel of prognostic markers in GC patients (Fig. 1) (Table 1).

**Table 1:** All of the miRNAs associated with cisplatin (CDDP) resistance in gastric tumor cells

Gene	**Target**	**Effect on the target**	**Effect on the tumor cells**	**Study**	**Year**
Signaling pathways					
Circ-AKT3	miR-198	Down regulation	Increased CDDP resistance	Huang [6]	2019
miR-34a	MET	Down regulation	Increased CDDP sensitivity	Zhang [7]	2016
miR-106a	PTEN	Down regulation	Increased CDDP resistance	Fang [15]	2013
miR-21	PTEN	Down regulation	Increased CDDP resistance	Yang [16]	2013
miR-195	AKT3	Down regulation	Increased CDDP sensitivity	Ye [17]	2017
CRAL	miR-505	Down regulation	Increased CDDP sensitivity	Wang [18]	2020
miR-7	mTOR	Down regulation	Increased CDDP sensitivity	Xu [19]	2017
miR-375	ERBB2	Down regulation	Increased CDDP sensitivity	Zhou [20]	2016
miR-101	VEGF-C	Down regulation	Increased CDDP sensitivity	Li [21]	2016
miR-362	CYLD	Down regulation	Increased CDDP resistance	Xia [22]	2014
miR-20a	CYLD	Down regulation	Increased CDDP resistance	Zhu [23]	2016
miR-20a	NFKBIB	Down regulation	Increased CDDP resistance	Du [24]	2016
miR-130a	CCL22	Down regulation	Increased CDDP sensitivity	Fang [25]	2020
miR-206	MAPK3	Down regulation	Increased CDDP sensitivity	Chen [26]	2019
miR-129	MAPK3	Down regulation	Increased CDDP sensitivity	Cao [27]	2019
Autophagy and apoptosis					
miR-181a	ATG5	Down regulation	Increased CDDP sensitivity	Zhao [28]	2016
MALAT1	miR-30b	Down regulation	Increased CDDP resistance	Xi [29]	2019
miR-218	BIRC5	Down regulation	Increased CDDP sensitivity	Zhang [30]	2018
Circ-CCDC66	miR-618	Down regulation	Increased CDDP resistance	Zhang [31]	2020
miR-136	AEG1, BCL2	Down regulation	Increased CDDP sensitivity	Yu [32]	2018
miR-449a	BCL2, CCND1	Down regulation	Increased CDDP sensitivity	Hu [33]	2014
miR-143	IGF1R and BCL2	Down regulation	Increased CDDP sensitivity	Zhuang [34]	2015
miR-148a-3p	RAB12, AKAP1	Down regulation	Increased CDDP sensitivity	Li [35]	2017
miR-193a-3p	SRSF2	Down regulation	Increased CDDP resistance	Lee [36]	2019
Epithelial Mesenchymal Transition (EMT)					
miR-200c	ZEB2	Down regulation	Increased CDDP sensitivity	Jiang [37]	2017
PCAT-1	miR-128	Down regulation	Increased CDDP resistance	Guo [38]	2019
miR-574-3p	ZEB1	Down regulation	Increased CDDP sensitivity	Wang [39]	2019
miR-30a	SNAI1, VIM	Down regulation	Increased CDDP sensitivity	Wang [40]	2016
miR-17	DEDD	Down regulation	Increased CDDP resistance	Wu [41]	2018
Drug efflux					
miR-129	MDR1	Down regulation	Increased CDDP sensitivity	Lu [42]	2017
miR-30a	MDR1	Down regulation	Increased CDDP sensitivity	Du [43]	2018
HOTAIR	miR-126	Down regulation	Increased CDDP resistance	Yan [44]	2016
HOTAIR	miR-34a	Down regulation	Increased CDDP resistance	Cheng [45]	2018
miR-132	SIRT1	Down regulation	Increased CDDP resistance	Zhang [46]	2017
Protein kinases					
Let-7b	AURKB	Down regulation	Increased CDDP sensitivity	Han [47]	2018
Hsa-Circ-0081143	miR-646	Down regulation	Increased CDDP resistance	Xue [48]	2019
miR-4290	PDK1	Down regulation	Increased CDDP sensitivity	Qian [49]	2020
miR-221-5p	DDR1	Down regulation	Increased CDDP sensitivity	Jiang [50]	2020
Transcription factors					
miR-613	SOX9	Down regulation	Increased CDDP sensitivity	Xue [51]	2019
miR-421	CASP3 and CDH1	Down regulation	Increased CDDP resistance	Ge [52]	2016
Circ-DONSON	miR-802	Down regulation	Increased CDDP resistance	Liu [53]	2020
Structural and DNA repair factors					
miR-101	ANXA2	Down regulation	Increased CDDP sensitivity	Bao [54]	2017
miR-223	FBXW7	Down regulation	Increased CDDP resistance	Zhou [55]	2015
miR-320a	ADAM10	Down regulation	Increased CDDP sensitivity	Ge [56]	2017
miR-876-3p	TMED3	Down regulation	Increased CDDP sensitivity	Peng [57]	2019
HOTTIP	miR-218	Down regulation	Increased CDDP resistance	Wang [58]	2019
miR-138-5p	ERCC1 and ERCC4	Down regulation	Increased CDDP sensitivity	Ning [59]	2019
Circ-0026359	miR-1200	Down regulation	Increased CDDP resistance	Zhang [60]	2020

### Signaling pathways

**Fig. 2:**
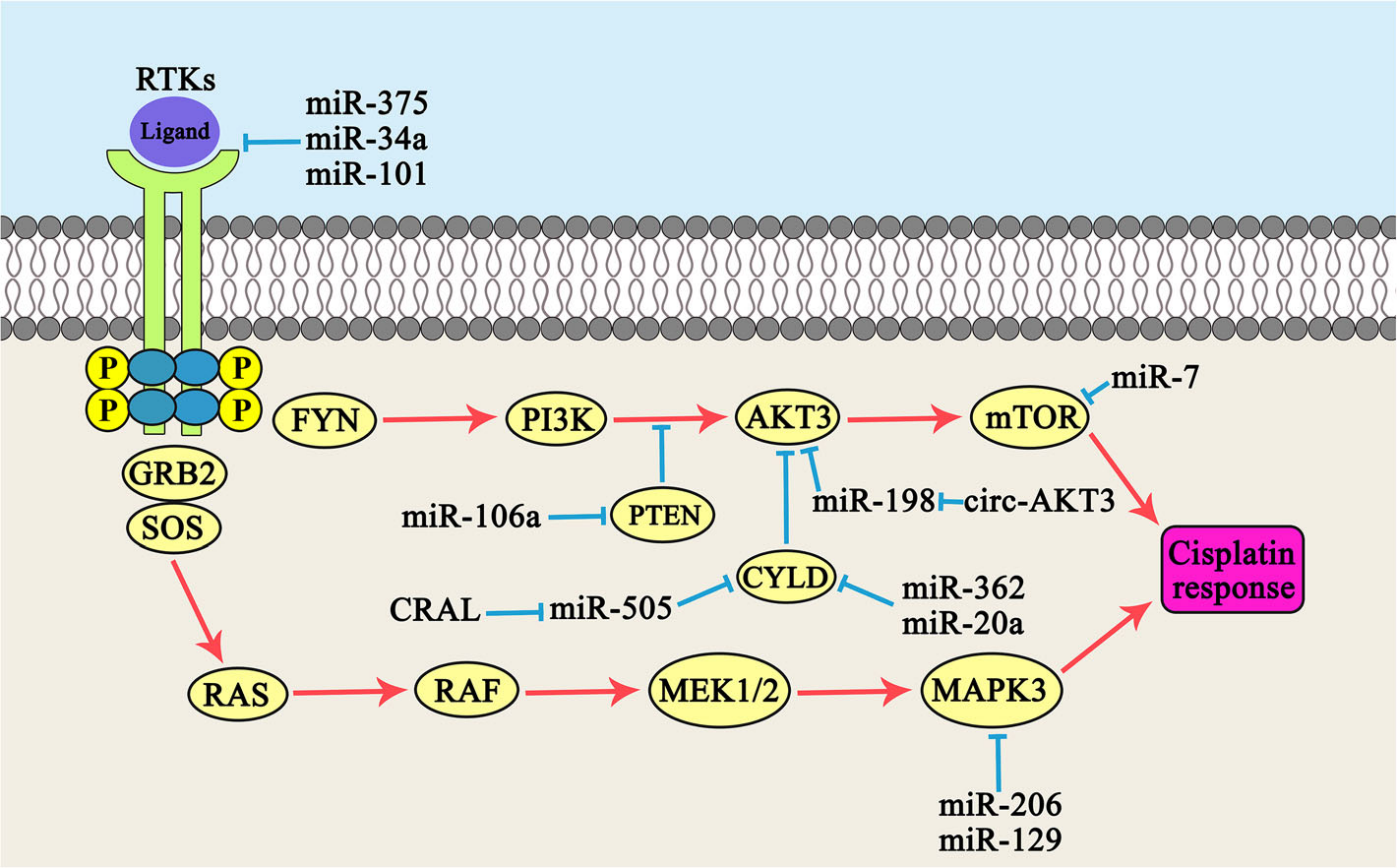
Role of miRNAs in regulation of cisplatin response in gastric tumor cells via MAPK and PI3K/AKT signaling pathways

MiRNAs are involved in regulation of cisplatin response in gastric tumor cells via MAPK and PI3K/AKT signaling pathways (Fig. 2). The PI3K/AKT is a critical cell survival signaling pathway that promotes apoptosis resistance [61, 62]. PTEN is a tumor suppressor that negatively modulates the activity of PI3K/AKT pathway and is also implicated in tumorigenesis and chemo resistance [63]. PTEN inhibits the activity of PI3K through PIP3 dephosphorylation [64]. An association was observed between the miR-106a ectopic expression and the resultant down regulation of PTEN which suggested the role of aberrant miR-106a expression in cisplatin-resistance of SGC7901/DDP cells. The findings also indicated that cisplatin-resistant SGC7901/DDP cells had significantly higher miR-106a expression levels as compared to parental SGC7901 cells [15]. It has been observed that there was a significant miR-21 up regulation in CDDP-resistant GC cells compared with their parental. MiR-21 increased CDDP resistance via PTEN targeting through the activation of PI3K/AKT pathway [16]. Long noncoding RNAs (lncRNAs) are a group of noncoding RNA longer than 200 bp that can be oncogene or tumor suppressor [65, 66]. They function as decoys for miRNAs or proteins [67]. LncRNAs act as inhibitors of miRNAs via absorbing and suppressing the miRNAs from binding to their target mRNAs in a process that is known as sponging. Cancer susceptibility candidate 2 (CASC2) is a lncRNA that increased CDDP sensitivity by miR-21 sponging and PTEN up regulation [68]. It has been shown that there were significant CASC2 down regulations in CDDP resistant GC tissues and cells. CASC2 increased CDDP sensitivity through miR-19a sponging in GC [69]. AKT is a serine/threonine kinase and the primary downstream effector of PI3K signaling which is involved in tumor progression and disease-free survival [70, 71]. The miR-195 exerts its suppressive effects on proliferation, invasion, and migration of GC cells via AKT3 targeting and suppression of PI3K/AKT pathway. It also increased cisplatin sensitivity in GC cells. Patients with a high miR-195 expression level had significantly longer progression-free survival (PFS) compared with those with miR-195 under expression [17]. MiR-34a enhanced cisplatin-sensitivity in GC cells through modulating PI3K/AKT/BIRC5 pathway [72]. Cylindromatosis (CYLD) functions as a deubiquitinating enzyme and exerts a tumor-suppressive role in multiple malignancies [73, 74]. CYLD is the negative modulator of PI3K/AKT/NF-κB axis and is also implicated in regulation of tumor cell apoptosis [75, 76]. The suppression of PI3K/AKT signaling pathway is effective in attenuating the chemo-resistance of GC cells [77, 78]. It has been observed that Cisplatin Resistance-Associated lncRNA (CRAL) up regulated CYLD and inhibited PI3K/AKT pathway through miR-505 targeting that regulated cisplatin response in GC cells [18]. mTOR belongs to the PIKKs family of serine/threonine protein kinases and is one of the effectors of PI3K/AKT pathway. It has key roles in anabolic cell metabolism through enhancing mRNA translocation and protein synthesis and is also implicated in glucose metabolism and lipid biosynthesis [79, 80]. It was found that gastric cancer tissues had significantly lower levels of miR-7 expressions compared to normal margins. MiR-7 suppressed GC cell proliferation and invasion through targeting mTOR. It also attenuated cisplatin-resistance via suppressing mTOR in GC cells [19].

Circular RNAs (circRNAs) are a group of non-coding RNAs (ncRNAs) forming a covalently closed continuous loop that lacks the 5’ and 3’ terminal nucleotide sequences. CircRNAs are implicated in transcriptional and post-transcriptional regulations [81]. CircRNAs can sponge miRNAs and inhibit their activity through functioning as competitive endogenous RNAs (ceRNAs). As a result, they affects different biological processes that are regulated by miRNAs. The inhibition of miRNAs by circRNAs suggests a novel mechanism of how miRNA’s activity is modulated and extends our understandings of circRNAs mode of action [81]. Activation of the PI3K/AKT signaling pathway enhances cell viability and inhibits apoptosis; therefore, this pathway plays a critical role in the chemo resistance of tumor cells [82, 83]. Induction of PI3K/AKT pathway prevents CASP3 activation by promoting its phosphorylation, thereby suppresses cell apoptosis [84]. It has been observed that there was significant circ-AKT3 up regulation in cisplatin-resistant compared to cisplatin-sensitive GC tissues and cells. Circ-AKT3 facilitated cisplatin-resistance in GC cells via sponging miR-198 and activating the PI3K/AKT signaling pathway [6]. ERBB2 belongs to the epidermal growth factor receptor family and a positive regulator of tumor cell proliferation [85]. Since, PI3K/AKT signaling is implicated in regulation of ERBB2; the induction of ERBB2/PI3K/AKT axis is correlated with chemo resistance [86]. There was a significant miR-375 down regulation in SGC7901/DDP cells compared to parental SGC7901 cells. The protein levels of both ERBB2 and p-AKT were suppressed in response to miR-375 overexpression. Therefore, it was hypothesized that miR-375 enhanced SGC7901/DDP cisplatin-sensitivity via targeting ERBB2/PI3K/AKT axis [20]. VEGF-C is a growth factor belonging to the VEGF family that promotes angiogenesis along with permeability of blood vessels via direct activation of VEGFR2/3 [87]. VEGF-C/VEGFR3 are pivotal regulators of lymphendothelial function by PI3K/AKT pathway [88]. There was significant miR-101 down regulation in GC cell lines in comparison with normal gastric epithelial cells. MiR-101 reduced cell proliferation while increased cisplatin-induced apoptosis in SGC7901/DDP cells via targeting VEGF-C [21].

NF-κB signaling inhibits apoptosis via transcriptional regulation of apoptotic related genes such as TRAF1, TRAF2, c-IAP1, and BCL-2 [89]. NF-κB is a heterodimer protein complex consisting of p65 and p50 subunits which are sequestered by a family of inhibitors called IκBs in the cytoplasm of unstimulated cells. Following the signal-induced degradation of IκB proteins, the NF-κB complex is activated and enters the nucleus to stimulate the transcription of multiple target genes. CYLD is a tumor suppressor deubiquitinating enzyme that functions as an inverse modulator of the NFκB signaling pathway and is implicated in the regulation of tumor cell apoptosis [90]. NFκB inhibition promotes chemo sensitivity in GC cells [22, 91]. BIRC5 and Livin are the members of inhibitor of apoptosis (IAP) family that inhibit caspase activation. It has been shown that there was a significant miR-20a up regulation in cisplatin resistant GC plasma and tissue samples. MiR-30a potentially was contributed to cisplatin resistance in GC through down regulating CYLD which leads to NFκB activation and up regulation of the downstream targets such as BIRC5 and Livin [23]. MiR-362 over expression was observed in gastric tumor tissues and cell lines. The results showed that miR-362 promoted the gastric tumor cell proliferation and resistance to cisplatin-induced apoptosis. Furthermore, miR-362 induced the activity of the NF-κB pathway in GC cells via targeting and down regulating CYLD [22]. NFKB inhibitor beta (NFKBIB) belongs to the NFκB inhibitor family [92]. Persistent activation of NF-κB and over expression of drug resistance-related proteins such as MDR1, MCL-1, BCL-2, and XIAP have critical functions in mediating chemo-resistance in various tumors [93–95]. Survivin and livin are two anti-apoptotic proteins belonging to the inhibitor of apoptosis (IAP) family which block caspase activity and inhibit cell apoptosis [96]. It has been observed that there was significant miR-20a up regulation in GC plasma and tissue samples with cisplatin-resistance. MiR-20a promoted cisplatin-resistance of GC cells through NFκB activation and up regulations of survivin and livin following NFKBIB targeting [24]. Chemokines are small molecules involved in regulation of various cellular processes through binding with G-protein-coupled receptors [97]. CCL22 is a member of the CC chemokine family which is secreted by dendritic cells and macrophages and is linked with tumor immune infiltration via CCR4 receptor [98, 99]. Chemokine receptor CCR4 promotes tumor invasion by activating ERK/NF-κB/MMP13 axis [100]. It decreases anti-tumor immunity in GC, while enhances the immunosuppression of regulatory T lymphocytes, and contributes to the CRC chemo-resistance [101, 102]. It has been reported that miR-130a-5p promoted cisplatin-sensitivity and suppressed malignant progression of the gastric tumor through CCL22 inhibition [25].

The ERK/MAPK signaling pathway is involved in regulation of multiple physiological and pathological processes such as cell proliferation, apoptosis, tumorigenesis, and chemo resistance [103–105]. MAPK is a serine/threonine kinase, which acts in the nuclear translocation of cytosolic proteins through phosphorylation, thereby regulating the functions of nuclear C-fos or C- transcription factors via phosphorylation [106, 107]. There was a significant MAPK3 up regulation in GC tissues compared with normal margins. MiR-206 reduced cisplatin-resistance in GC cells through MAPK3 targeting. It also decreased cell proliferation and increased apoptosis in GC cells [26]. MiR-129 reduced cell proliferation and CDDP resistance via MAPK3 targeting in GC cells [27]. Transforming growth factor beta (TGFB) signaling is involved in embryogenesis, cell differentiation, and apoptosis via activation of SMAD protein complexes that act as transcription factors to modulate the expression of target genes. There was an inverse association between the levels of miR-187 expression and cisplatin resistance in GC cells. The up regulation of miR-187 suppressed the expression of TGF-β1, p-Smad4, ERCC3, and ERCC4 in GC cells implying the role of the TGF-β/Smad axis in miR-187-regulated cisplatin-resistance [108].

### Autophagy and apoptosis

**Fig. 3:**
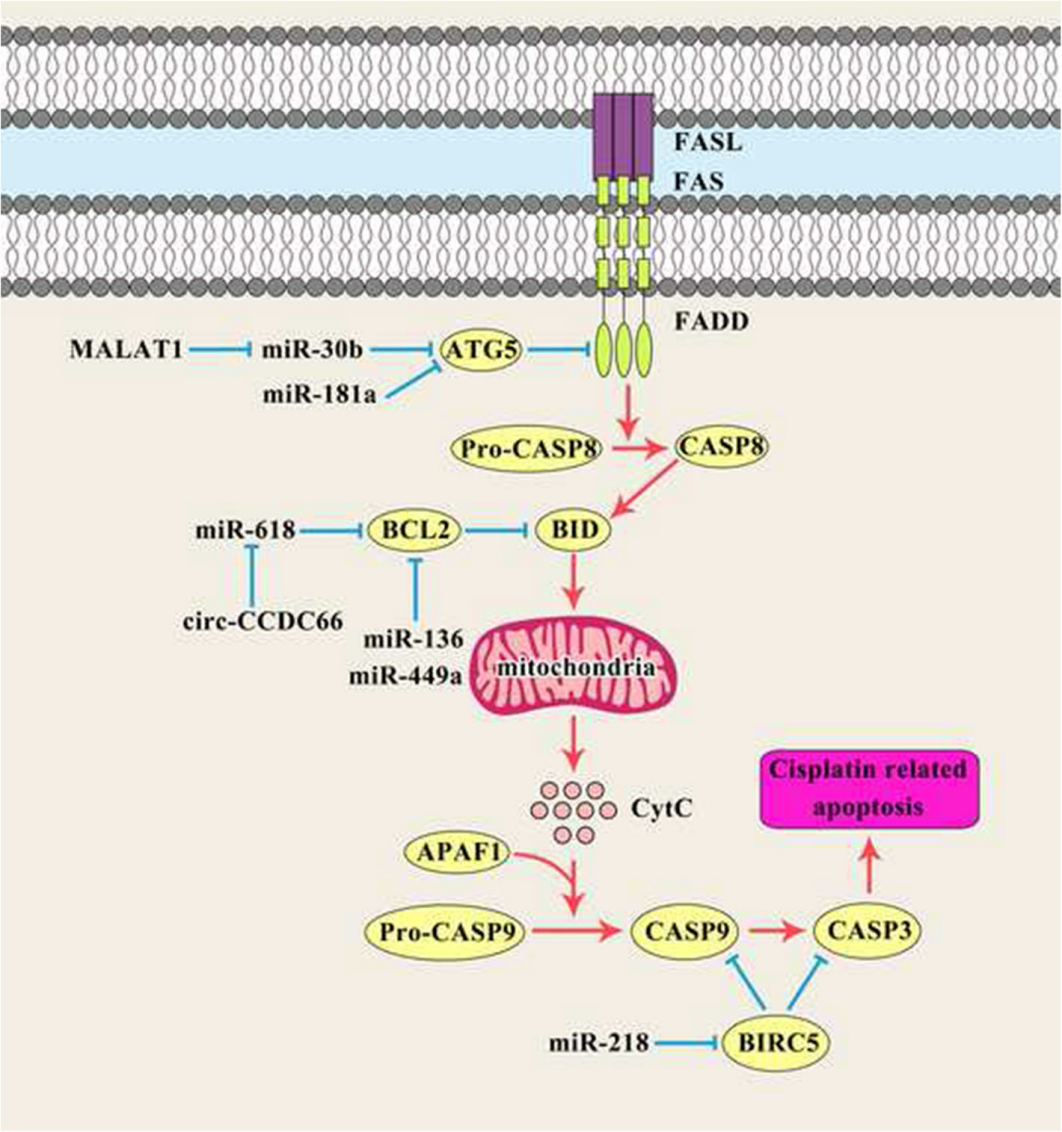
miRNAs are involved in cisplatin response of gastric tumor cell by regulation of apoptosis and autophagy

Autophagy is an intracellular self-digesting process for the regulation of cell homeostasis during which malformed or excessive proteins and long-lived or damaged organelles are removed by lysosomes [109]. While the majority of studies indicate that autophagy allows prolonged survival [110, 111], some others show that the autophagy enhances autophagic cell death [112]. Therefore, suppression of autophagy offers an efficient combined therapeutic approach for the regulation of chemo sensitivity in cancer. It has been reported that autophagy exerts oncogenic or tumor-suppressive functions during GC progression [113]. Cisplatin chemotherapy is believed to promote autophagy in some human malignancies [114, 115]. MiRNAs are involved in cisplatin response of gastric tumor cells by regulation of apoptosis and autophagy (Fig. 3). MiR-181a suppresses autophagy in cisplatin-resistant GC cells via ATG5 targeting [28]. MALAT1 and ATG5 improve chemo-resistance of cancer cells through increasing autophagy [116, 117]. It has been observed that there was significant MALAT1 up regulation in cisplatin-resistant GC cells. MALAT1 increased cisplatin-resistance via enhancing autophagic activity in AGS/CDDP gastric tumor cells. MiR-30b attenuated autophagy-related cisplatin-resistance through targeting ATG5 in cisplatin-resistant gastric tumor cells. MALAT1 Up regulated ATG5 in cisplatin-resistant GC cells via functioning as a ceRNA for miR-30b to sequester miR-30b from ATG5 [29].

Survivin belongs to the IAP family and is encoded by the BIRC5 gene [118]. Survivin exerts its pro-survival functions via inhibiting the activity of caspases [119]. It has been shown that survivin form a complex with hepatitis B X-interacting protein to regulate the activation of CASP9 [120]. Studies have also highlighted the role of survivin in anti-cancer chemotherapy resistance and have introduced survivin as a putative biomarker for predicting chemo resistance [120, 121]. It has been reported that there was a significant miR-218 down regulation in cisplatin-resistant SGC7901/DDP gastric tumor cells. MiR-218 promoted cisplatin-sensitivity in SGC7901/DDP cells via BIRC5 inhibition [30]. BCL2 is a negative regulator of apoptosis [122]. It has been reported that there was circCCDC66 up regulation in CDDP-resistant GC patients. Circ-CCDC66 induced CDDC resistance via miR-618 inhibition that resulted in BCL2 up regulation in gastric tumor cells [31]. MiR-136 and miR-449a also increased CDDP sensitivity of gastric tumor cells through BCL2 targeting [32, 33]. There was a significant miR-143 down regulation in cisplatin-resistant GC cell line. MiR-143 regulated cisplatin-resistance through IGF1R and BCL2 targeting. Cisplatin-resistant GC cells transfected with miR-143 mimics had reduced cell proliferation, while increased apoptosis [34]. Mitochondria function in energy production and also play key roles in cancer metabolic homeostasis [123]. Following exposure to anti-cancer drugs, the intrinsic apoptotic pathway is activated and leads to cancer cell death [124]. Mitochondrial fission is a crucial stage in the initiation of mitochondria-dependent apoptosis [125]. Dynamin-Related Protein 1 (DRP1) is considered as an upstream modulator of mitochondrial fission which controls and accomplishes the final part of mitochondrial fission [126]. Mitochondrial fission 1 protein (FIS1) functions as a receptor for recruiting DRP1 to mitochondria that is indirectly involved in mitochondrial fission. It has been demonstrated that FIS1 is implicated in CDDP-sensitivity in tongue squamous cell carcinoma [127]. AKAP1 functions as a scaffold that presents PKA to downstream targets at the mitochondrial membrane to regulate their phosphorylation state [128]. RAB12 belongs to the Ras family of oncogenes and is involved in the induction of autophagy through speeding up autolysosome maturation or suppression of mTORC1 activity [129, 130]. There were miR-148a-3p down regulations in cisplatin-resistant GC tissue and cell lines. MiR-148a-3p enhances cisplatin cytotoxicity in gastric tumor cells through targeted inhibition of RAB12 and AKAP1. In response to cisplatin treatment, miR-148a-3p promoted mitochondrial fission-induced apoptosis via targeting AKAP1 and increasing P53 and DRP1 activation [35]. SRSF2 belongs to the serine/arginine-rich protein family that is critical for splice-site selection during the alternative splicing process of mRNA precursors. CD44 is a GC stem cell marker that plays key roles in modulating tumorigenesis, self-renewal, distant metastasis, and chemo-resistance. It has been reported that miR-193a-3p was positively correlated with cisplatin resistance in CD44 + GC cells. There was inverse correlation between the levels of miR-193a-3p and SRSF2 expressions. CD44 + GC cells had high levels of BCL-2 expression, while low levels of CYCS, BAX, CASP3, and CASP9 expressions compared with CD44- cells. MiR-193a-3p suppressed CDDP-induced mitochondrial cell death in CD44 + GC cells [36].

### Epithelial-mesenchymal transition

Epithelial-mesenchymal transition (EMT) is a cellular process in which the epithelial cells lose their cell adhesion and polarity to convert into the invasive mesenchymal cells. It is a pivotal cellular mechanism during tumor growth, metastasis, and chemo resistance [131–134]. The expressions of epithelial genes such as CDH1, ZO-1, and OCLN are suppressed during EMT; while, mesenchymal markers including CDH2, α-SMA, FN, and VIM are overexpressed during this process [135, 136]. The initiation of EMT occurs following the up regulation of its related transcription factors such as SNAI1, TWIST1, and ZEB1 [137]. ZEB2 is a key transcription factor that belongs to the Snail superfamily and is normally expressed in human tissues. ZEB2 suppresses the transcription of CDH1, cytokeratin, MAC1, and mucin proteins through binding to the E-box sequence in the promoter region of target genes. Low expression levels of the above-mentioned proteins are associated with the EMT [138]. ZEB2 expression is also associated with resistance to cisplatin and EGFR inhibitors in ovarian and bladder cancers, respectively [139, 140]. It has been observed that there was significant miR-200c down regulation in GC tissues and SGC7901/DDP cells in comparison with normal margins and parental SGC7901 cells. MiR-200c sensitized GC cells to cisplatin treatment via ZEB2 targeting [37]. There was PCAT-1 up regulation in CDDP-resistant gastric tumor cells. PCAT-1 increased CDDP resistance by of GC cells through miR-128 sponging that resulted in increased levels of ZEB1 expression in gastric tumor cells [38]. It has been observed that miR-574-3p suppressed EMT via ZEB1 targeting. MiR-574-3p also suppressed cisplatin resistance in GC cells both in vivo and in vitro [39]. Another study have reported that there was a significant miR-30a down regulation in cisplatin-resistant GC compared with sensitive tissues. Cisplatin-resistant (SGC7901/DDP) cells also showed significantly lower miR-30a expression levels than the cisplatin-sensitive SGC7901 cells. Morphological features were also different between SGC7901/DDP (extended fibroblastoid-like) and SGC7901 (epithelial-like) cell lines. There were SNAI1 and VIM up regulations in SGC-7901/DDP cells in comparison with SGC-7901 cells. MiR-30a suppressed SNAI1 and VIM expressions and induced morphological changes from an elongated fibroblastoid-like to an epithelial-like morphology in SGC-7901/DDP cells [40]. Death effector domain-containing protein (DEDD) plays important roles in multiple cellular processes such as cell cycle, mitosis, and apoptosis [141]. There is a correlation between EMT and DEDD in various cancers [136, 142]. The findings revealed that miR-17 enhanced EMT and cisplatin-resistance through targeting DEDD in GC cells [41].

### Drug efflux

There are various mechanisms involved in development of multidrug resistance (MDR) in tumor cells including DNA repair ability, cell cycle regulation, drug efflux/uptake, and detoxification agents [143–145]. P-glycoprotein (P-gp) is a trans membrane protein encoded by the MDR1 gene that is widely expressed in normal tissues [146]. P-gp is an important ATP-dependent drug efflux transporter playing a critical role in tissue homeostasis, detoxification, and protection against hazardous metabolites. Up regulation of P-gp in tumor cells occurs following exposure to chemotherapy that is observed in almost 30 % of cancer cases [147]. It has been reported that miR-129 down regulated P-gp and activated intrinsic apoptosis pathway via the over expressions of CASP9 and CASP3. It was also found that miR-129 was markedly down regulated in cisplatin-resistant vs. cisplatin-sensitive clinical specimens. MiR-129 reduced cisplatin resistance of GC cells through targeting P-gp [42]. There was a significant miR-30a down regulation in SGC7901/CDDP cells. MiR-30a exerted its inhibitory functions on tumor cell proliferation and cisplatin-resistance via MDR1 targeting [43]. HOTAIR is a lncRNA transcribed from the homeobox C (HOXC) gene [148]. It has been reported that the up regulation of HOTAIR was associated with chemo resistance in various cancers through different mechanisms. HOTAIR promotes chemo resistance of ovarian cancer via WNT pathway activation [149]. It also contributes to tamoxifen resistance in breast cancer through inducing estrogen receptor (ER) signaling [150]. It has been shown that there were HOTAIR up regulations in GC tissues and cisplatin-resistant GC cells compared with normal margins and control cells. HOTAIR promoted resistance to cisplatin treatment in GC cells via miR-126 sponging that resulted in increased activity of PI3K/AKT/MRP1 axis [44]. HOTAIR up regulation was observed in GC tissues and cells compared with normal margins and cell lines. There was also an inverse correlation between the levels of HOTAIR and miR-34a expressions in GC tissues. HOTAIR knockdown suppressed cisplatin-resistance in gastric tumor cells through miR-34a up regulation [45]. Cancer stem cells (CSCs) are a subpopulation of highly oncogenic and chemo resistant tumor cells that have an important role in tumor progression and recurrence [151]. It has been reported that Lgr5 + GC stem cells (GCSCs) had significantly higher levels of miR-132 compared with Lrg5- cells. MiR-132 also increased cisplatin-resistance in Lgr5 + GCSCs both in vitro and in vivo. There was an inverse correlation between the levels of SIRT1 and miR-132 expressions in GC samples. Silencing of SIRT1 led to the up regulation of ABCG2 through the induction of CREB acetylation [46].

### Protein kinases

Aurora kinase B (AURKB) belongs to the serine/threonine protein kinases that is implicated in regulation of mitosis and chromosome segregation [152]. AURKB phosphorylates VIM to regulate vimentin filament segregation during cytokinesis. Patients with up regulation of AURKB experience higher overall survival rates compared with those with AURKB down regulation [153]. There was a significant let-7b down regulation in cisplatin-resistant GC cells compared with parental cells. Let-7b induced sensitivity to cisplatin and suppressed tumor growth through targeting AURKB [47]. CDK6 belongs to the cyclin-dependent kinase (CDK) family and is implicated in different biological and pathological processes including cell cycle progression, centrosome stability, apoptosis, angiogenesis, and chemo-resistance [154, 155]. It was found that hsa-circ-0081143 enhanced gastric tumor cell proliferation and chemo-resistance through miR-646/CDK6/KLF5 axis. Hsa-circ-0081143 was significantly overexpressed in GC patients which was associated with higher tumor, nodes, metastases (TNM) stage and poorer prognosis. Inhibition of hsa-circ-0081143 suppressed GC progression, induced apoptosis, and attenuated their cisplatin resistance. There was a significant inverse association between the miR-646 and hsa-circ-0081143 expressions. The hsa-circ-0081143 positively regulated CDK6 expression through targeting miR-646 [48]. In contrast with the normal cells which tend to shift to the aerobic respiration pathway under oxygen availability, tumor cells boost their glucose uptake and continue to undergo glycolysis. Dysregulation of glycolysis is implicated in chemo-resistance [156]. Pyruvate dehydrogenase kinase 1 (PDK1) as a major glycolytic enzyme, inhibits pyruvate oxidation through inactivation of pyruvate dehydrogenase [157]. There was a miR-4290 down regulation in GC tissues which was correlated with advanced TNM stage and poor prognosis. MiR-4290 inhibited glycolysis through directly targeting PDK1 that increased cisplatin sensitivity in GC cells [49]. Receptor tyrosine kinases (RTKs) are activated by growth factors, hormones, and cytokines. They are also key factors during tumor progression and metastasis. Discoidin domain receptor 1 (DDR1) is a RTK which is up regulated in multiple human cancers and is a putative regulator of various biological processes in tumor cells [158, 159]. It functions as a collagen receptor to regulate the ECM remodeling, cell migration, and proliferation via MAPK signaling pathway. DDR1 regulates ECM remodeling by MMPs up regulations to increase cell migration. DDR1 over expression was correlated with unfavorable prognoses [160]. It has been observed that there were significant miR-221-5p down regulations in GC tissues and cells. MiR-221-5p promoted apoptosis and cisplatin-sensitivity, while inhibited gastric tumor cell proliferation and migration through DDR1 targeting [50]. MET is also another RTK that is activated by hepatocyte growth factor (HGF) to regulate cell proliferation and survival. Activated MET interacts with various cytoplasmic downstream proteins such as PLCG1 and SRC which resulted in activation of RAS/ERK and PI3K/AKT signaling pathways. It has been reported that there was significant miR-34a down regulation in cisplatin-resistant GC tissues and cells. MiR-34a reduced cell proliferation, while promoted apoptosis of cisplatin-resistant GC cells by MET targeting [7].

### Transcription factors

Forkhead box O3a (FOXO3a) is a transcription factor involved in regulation of cell cycle progression, autophagy, and apoptosis [161, 162]. FOXO3a suppresses cell cycle progression via p27Kip1 regulation [163]. There was a significant miR-25 up regulation in cisplatin-resistant GC cells. MiR-25 was associated with reduced sensitivity of SGC-7901 cells to cisplatin treatment by FOXO3a targeting. MiR-25 silencing in cisplatin-resistant SGC-7901/DDP cells led to cell cycle arrest through p27Kip1 down regulation [164]. SOX9 is a developmental transcription factor widely expressed in various embryonic tissues [165, 166]. There were miR-613 down regulations in GC tissues and cell lines. MiR-613 sensitized GC cells to cisplatin, while inhibited cell proliferation, cell cycle progression, and migration through SOX9 targeting [51]. Hypoxia is a frequent process in tumor cells that is associated with pathological processes such as tumorigenesis, angiogenesis, migration, and chemo resistance [167, 168]. HIF-1α is regarded as the main transcription factor involved in regulation of signaling pathways related to cell cycle, apoptosis, and metastasis that results in transformation of cancer cells to a more malignant phenotype under hypoxic conditions [169]. Up regulation of miR-421 in gastric tumor cells suppresses apoptosis, enhances metastasis, and promotes cisplatin-resistance via CASP3 and CDH1 targeting. HIF-1α-induced-miR-421 is implicated in modulation of chemo-resistance and apoptosis suppression. MiR-421 up regulated CDH2, VIM, SNAI1, Twist, SNAI2, MMP-2, while inhibited CDH1 in GC cells [52]. BMI1 is a component of polycomb repressor complex 1 (PRC1) that functions as an oncogene through modulating the cell proliferation, apoptosis, and invasion of tumor cells. Deregulation of BMI1 has been associated with tumorigenesis and chemo resistance in multiple malignancies [170, 171]. It has been reported that there was circDONSON up regulation in cisplatin-resistant GC tissues and cell lines. CircDONSON knockdown enhanced tumor cell apoptosis, reduced cell viability, and attenuated cisplatin-resistance in vitro. CircDONSON indirectly regulated the BMI1 expression levels via sponging miR-802 in GC cells [53].

### Structural and DNA repair factors

Annexin A2 (ANXA2) is a calcium-dependent phospholipid-binding membrane protein involved in linkage of protein complexes to the cytoskeletal components [172]. It also regulates cell proliferation, differentiation, and migration in different cancers [173, 174]. It has been shown that there were miR-101 down regulations in GC cells and tissues. MiR-101 increased cisplatin sensitivity of GC cells through ANXA2 targeting [54]. FBXW7 is the substrate detection component of a SCF complex that mediates degradation of various target proteins such as NOTCH1, NOTCH2, and JNK [175, 176]. MiR-223 enhanced cisplatin-resistance of GC by targeting FBXW7 [55]. ADAM metallopeptidase domain 10 (ADAM10) is a metalloproteinase that is frequently up regulated in human tumors which triggers cancer initiation, progression, and metastasis [177, 178]. ADAM10 expression in non-small cell lung cancer and pituitary adenoma contributes to cell migration through activating the NOTCH signaling pathway and regulating the cleavage of CD44, respectively [179, 180]. Moreover, the expression of ADAM10 is correlated with the invasive behavior of cancer cells in pancreatic carcinoma and oral squamous cell carcinoma [181, 182]. It has been reported that there were miR-320 down regulations in GC tissues and cells. MiR-320a enhanced cisplatin-sensitivity in GC cells through ADAM10 suppression [56]. TMED3 is a trans-membrane protein involved in vesicular protein trafficking and Golgi protein modification. There were positive correlations between miR-876-3p down regulation and clinicopathological features including poorer prognoses and shorter overall survival rates of GC patients. MiR-876-3p modulated cisplatin sensitivity and stem cell-like morphology and properties of gastric tumor cells via targeting TMED3. Down regulation of miR-876-3p was more prominent in chemo-resistant than chemo-sensitive GC tissues [57]. HMGA1 is a chromatin-associated protein involved in regulation gene expression. There was HOTTIP up regulation in cisplatin-resistant GC cells. HOTTIP promoted cisplatin resistance through activating HMGA1 via functioning as a ceRNA for miR-218 [58]. ERCC is a pivotal member of nuclear excision repair (NER) that is considered as the principal cause of cisplatin resistance in tumor cells. ERCC1 and ERCC4 heterodimer possesses a potential endonuclease activity to excise the 5’ side of DNA damage during NER process. MiR-138-5p regulates cisplatin-sensitivity of GC cells through ERCC1 and ERCC4 targeting [59]. POLD4 is a component of DNA polymerase delta that has pivotal roles in DNA replication and repair via regulation of POLD1 and 3’ to 5’ proofreading activity [183]. Gastric tumor tissues and cell lines had significant circ_0026359 up regulations compared with normal margins and cell lines. There were significant circ_0026359 up regulations in cisplatin resistant GC tissues and cell lines. Circ_0026359 up regulation was positively correlated with shorter overall and relapse-free survival rates in GC patients. Depletion of circ_0026359 attenuated cisplatin-resistance of gastric tumor cells through inducing miR-1200 activity and down regulating POLD4 [60].

### Perspective and hurdles

Direct assessment of tissue biomarkers improves tumor diagnosis, however, the invasive procedures required to obtain tumor biopsies limit their application. An alternative approach is to study miRNAs in body fluids, such as blood, which are also deregulated in cancer [184] and, furthermore, have the distinct advantage of requiring a much less invasive procedure for sample collection. Circulating miRNAs are also stable in different pH conditions, repeated freeze-thawing, and room temperature which support the miRNAs as diagnostic and prognostic molecular biomarkers [185, 186]. Regarding the small size and low concentration of circulating miRNAs in body fluids, accurate quantification should be done by various molecular techniques such as qRT-PCR, Microarray platforms, and Next-generation sequencing [187, 188]. Some of the miRNAs have been reported to affect anticancer drug response [189–191]. Although, single miRNA can be used to predict the chemotherapeutic response, a panel of miRNAs signature is more efficient. A signature of several miRNAs was associated with CDDP and 5-fluorouracil resistances in GC patients [192]. Therefore, drug responses can be affected by the manipulation of miRNA levels. The miRNA-based treatment is the suppression of deregulated miRNAs using miRNA sponges and anti-miRNA oligonucleotides [193]. Since, miRNAs affect the target mRNAs by partial sequence complementarity, the off-target effect results in unfavorable immune response and side effects. They are also sensitive toward the cellular nucleases that prevent the development of efficient miRNA-based therapies. Moreover, antimiRs may also negatively affect physiological functions that are normally regulated by the target miRNAs. Therefore, site specific, lowest optimum concentration, and delivery systems are pivotal factors to obtain the best therapeutic results with the lowest side effects in miRNA based treatment [194]. MiRNA mimics can be delivered by vectors, nanocarriers, and amphiphilic star copolymer [195–197]. Nanoparticle delivery of synthetic anti oncomiRs or synthetic tumor suppressor miRNAs in combination with chemotherapeutic drugs has been reported in cancer therapy [198]. It is required to clarify the molecular interactions of the miRNAs involved in regulation of CDDP response to introduce a prognostic miRNA panel marker for the prediction of CDDP response. For the first time, present review uncovers the molecular interactions of miRNAs during GC progression.

## Conclusions

Since, there is a high rate of recurrence after CDDP treatment in GC patients; it is required to clarify the molecular mechanisms associated with CDDP resistance to introduce novel therapeutic methods. MicroRNAs are a class of endogenous non-coding RNAs involved in chemo resistance of GC cells through regulation of all of the MDR mechanisms. In present review we have summarized all of the miRNAs associated with cisplatin resistance based on their target genes and molecular mechanisms in gastric tumor cells. This review paves the way of introducing a miRNA-based panel of prognostic markers to improve the efficacy of chemotherapy and clinical outcomes in GC patients. It was observed that miRNAs are mainly involved in cisplatin response of gastric tumor cells via regulation of signaling pathways, autophagy, and apoptosis.

## Data Availability

The datasets used and/or analyzed during the current study are available from the corresponding author on reasonable request.

## Abbreviations

GC: Gastric cancer
lncRNAs: Long noncoding RNAs
CASC2: Cancer susceptibility candidate 2
PFS: Progression-free survival
circRNAs: Circular RNAs
ncRNAs: Non-coding RNAs
ceRNAs: Competitive endogenous RNAs
CYLD: Cylindromatosis
NFKBIB: NFKB inhibitor beta
IAP: Inhibitor of apoptosis
TGFB: Transforming growth factor beta
DRP1: Dynamin-Related Protein 1
EMT: Epithelial-mesenchymal transition
DEDD: Death effector domain-containing protein
MDR: Multidrug resistance
P-gp: P-glycoprotein
HOXC: Homeobox C
ER: Estrogen receptor
CSCs: Cancer stem cell-like cells
GCSCs: GC stem cells
AURKB: Aurora kinase B
CDK: Cyclin-dependent kinase
PDK1: Pyruvate dehydrogenase kinase 1
RTKs: Receptor tyrosine kinases
DDR1: Discoidin domain receptor 1
HGF: Hepatocyte growth factor
FOXO3a: Forkhead box O3a
PRC1: Polycomb repressor complex 1
ANXA2: Annexin A2
ADAM10: ADAM metallopeptidase domain 10
NER: Nuclear excision repair
CRAL: Cisplatin Resistance-Associated lncRNA
TNM: Tumor, nodes, metastases
